# Fear and anxiety related to COVID-19 pandemic may predispose to perinatal depression in Italy

**DOI:** 10.3389/fpsyt.2022.977681

**Published:** 2022-08-03

**Authors:** Laura Orsolini, Simone Pompili, Antonella Mauro, Virginio Salvi, Umberto Volpe

**Affiliations:** Unit of Clinical Psychiatry, Department of Neurosciences/DIMSC, Polytechnic University of Marche, Ancona, Italy

**Keywords:** COVID-19, depression, peripartum, perinatal mental health, postpartum, pregnancy, women's mental health

## Abstract

The COVID-19 pandemic situation significantly affected the mental health of the general and clinical population. However, few studies investigated which COVID-19-related psychopathological determinants may predispose to perinatal depression. We evaluated the impact of COVID-19 related anxiety and fear on perinatal depression in Italy. We retrospectively screened 184 perinatal outpatients afferent to Perinatal Mental Health outpatient service, during March 2020-March 2021, by administering the Edinburgh Postnatal Depression Scale (EPDS), the Fear of COVID-19 (FCV-19-S) and the Coronavirus Anxiety Scale (CAS). Among these, 85 patients agreed to be recruited in the present study. The mean EPDS score was 9.0, experiencing a clinically relevant perinatal depression in 45.7% of the sample. The mean FCV-19-S score was 15.0 and CAS was 1.7. Linear regression analyses demonstrated that FCV-19-S and CAS scores statistically significantly predicted EPDS total scores. A positive significant correlation was reported between FCV-19-S and EPDS and between CAS and EPDS. During the COVID-19 pandemic, women in their perinatal period, independently of previous psychiatric history, experienced increased levels of anxiety, fear and psychological distress, due to subsequent isolation, quarantine, lockdown and deprivation of their normal social support. Further preventive and screening strategies should be implemented in order to early identify at-risk pregnant and puerperal women during the COVID-19 pandemic.

## Introduction

The COVID-19 pandemic situation significantly affected the mental health of the general and clinical population (1–5). The COVID-19-related situation determined a significant psychological distress, by determining increased levels of fear, anger and uncertainty, anxiety and depression symptomatology, suicidality, post-traumatic-related symptomatology, sleep disorders, and it facilitated the onset of *de novo* brief psychotic episodes, and so forth (6–15). Although few studies investigated the impact of COVID-19 pandemic and related restrictive measures on the women's mental health, during the pregnancy and the postpartum period, it was documented an overall increased incidence of anxious and depressive symptomatology in the perinatal period during the COVID-19 pandemic compared to pre-COVID-19 times (16–18).

The perinatal period (i.e., that period including all pregnancy and the first postpartum year) (19), indeed represents a critical vulnerable period for the *de novo* onset and recurrence of mental conditions, especially among women with a positive psychiatric history or those who experience gestational and/or delivery complications (20, 21). Based on the bio-psycho-social paradigm of mental disorders (22), the perinatal period may predispose women to experience high psychological distress due to physiological, biological, and social changes (17, 23–25). Moreover, within this framework, experiencing stressful and subjectively perceived traumatic events, during the perinatal period, may predispose women to the onset of *de novo* psychopathological manifestations, also in not predisposed pregnant and puerperal women (26). Therefore, one could argue that the COVID-19 pandemic and related restrictive measurements may have more likely represented a stressful and cumulative traumatic variable which might have modified the psychopathological trajectory in pregnancy and postpartum period, as already reported in the general population (27, 28) and in samples constituted by pregnant and postpartum women (18, 29–31). In fact, the gradual shaping in health care access and services due to the lockdown and restrictive regulations imposed by governments, including limitations in the access to gynecological, obstetrician and perinatal care and the restricted (or interrupted) possibility for partner and/or family member(s) of pregnant and puerperal women to assist them during pregnancy follow-ups, the delivery and postpartum period, significantly determined a psychological distress, an increased uncertainty and indeed fueled feelings of fears, anxiety and worries among pregnant and postpartum women (32–34). Moreover, perinatal women's mental health was also compromised by anxiety levels and worries related to disinformation overflow about COVID-19 pandemic and consequences for pregnant women's health and new-borns' health in case of COVID-19 infection during the pregnancy and/or early postpartum, as well as the uncertainty about the future (24, 35, 36).

Therefore, within the context of a multicenter nationwide population-based naturalistic observational project on perinatal depression, a retrospective chart-review study was carried out at the Unit of Clinical Psychiatry, Department of Neurosciences, University-Hospital “Ospedali Riuniti,” in Ancona, Italy, in collaboration with the Unit of Clinical Gynecology and Obstetrics, University Hospital “Salesi,” in Ancona, Italy. The main purpose of the larger observational protocol was to implement diagnostic and therapeutic interventions for early detection of at-risk women for occurring perinatal mental disorders as well as provide timely treatments. Within this larger project, our study firstly aimed at retrospectively analyzing those data collected during the COVID-19 pandemic, to evaluate the potential impact of COVID-19 related anxiety and fear on perinatal women's mental health, particularly perinatal depression levels. Given the exploratory nature of the study, we had no *a priori* hypothesis.

## Methods

### Study design and selection of participants

A retrospective chart-review study was carried out by recruiting all women afferent to the Peripartum Psychiatry Outpatient Service of the Unit of Clinical Psychiatry, at the University Hospital “Ospedali Riuniti,” Polytechnic University of Marche, Ancona, Italy, and/or hospitalized at the Unit of Clinical Gynecology and Obstetrics at the University Hospital “Salesi,” in Ancona, Italy, during the timeframe March 2020 to March 2021. Written informed consent was obtained from the patients after they were informed about the purpose of the study and the related methods. The study was introduced as aiming to assess whether pregnant or puerperal women's mental health changed during the Italian phase I-II-III of the COVID-19 pandemic and whether factors associated with the COVID-19 restrictions affected the course of perinatal symptomatology. Patients were retrospectively included in the study if they met the following inclusion criteria: (a) ≥18 years old; (b) education level not lower than elementary school; (c) absence of linguistic difficulties (i.e., not Italian speaker or foreign without a sufficient ability to understand Italian language); (d) no intellectual disability; (e) absence of severe medical conditions not related to the pregnancy and/or postpartum clinical situation; (f) pregnant women or within their first year of postpartum; (g) signed informed consent for collecting and analyzing clinical data for research purpose, collected during the baseline assessment. Participants were excluded if they met one or more of the following exclusion criteria: (a) intellectual disability or cognitive impairment; (b) diagnosis of organic mental disorder according to the DSM-5 criteria (37); (c) being under the influence of substances and/or alcohol; (d) incomplete filled out questionnaire; (e) refusal to participate to the research study. Recruited patients had also the possibility to withdraw their participation without any kind of clinical and therapeutic consequences. All procedures performed in studies involving human participants were in accordance with the ethical standards of the institutional and/or national research committee and with the 1964 Helsinki Declaration and its later amendments or comparable ethical standards. The Institutional Review Board approved our study. This research study was conducted retrospectively from data obtained for clinical purposes. All patients gave written consent to the use of clinical data for research purposes.

### Measures

An *ad hoc* case report form was specifically designed by the researchers to collect sociodemographic data (e.g., age, ethnic, marital status, housing condition, employment status, education level) and clinical data and pregnancy-related correlates (e.g., family context, social support, medical history, psychiatric personal and family history, number of children, obstetric-gynecologic variables, such as last menstruation date [LMD], estimated delivery date [EDD], previous history of miscarriages or induced abortion, delivery course and immediate outcomes).

As a screening tool for diagnosing pregnant and postpartum women who are at risk for perinatal depression, it was used the *Edinburgh Postnatal Depression Scale* (EPDS) (38–40). EPDS is a 10-items, four-point Likert-type self-assessment questionnaire, which was developed based on the American College of Obstetrics and Gynecology (ACOG) recommendations (41), to assess mood in pregnant women during the past week. The EPDS total score ranges from 0 to 30, with a clinically relevant cut-off ≥12 which indicates a higher risk for perinatal depression in the Italian sample (42, 43).

The following scales have been administered to evaluate the following COVID-19-related psychopathological dimensions: i.e., experiences of fear by using the Fear of COVID-19 Scale (FCV-19-S) (44, 45) and anxiety symptomatology by using the Coronavirus Anxiety Scale (CAS) (46–48). The FCV-19-S is a 7-items, 5-point Likert-type questionnaire (1= “strongly disagree”, 5= “strongly agree”), measuring the emotional fear occurring during COVID-19 pandemic. The total score ranged from 7 to 35, with a cut-off≥16.86 that was used to identify a significant risk of fear and other related disorders in the Italian sample (44, 45). The CAS is a 5-item, 5-point Likert-type self-report tool designed to measure the levels of dysfunctional anxiety related to the COVID-19 pandemic over the preceding 2 weeks, with a clinically relevant cut-off ≥9 in the Italian sample (46, 48).

### Statistical analysis

Statistical analyses were performed using SPSS (MACOS version 26; IBM Corp, Harmony [NY], 2019). Descriptive statistics were expressed as mean and standard deviation (SD) for the qualitative variables (EPDS, WDEQ, CAS and FCV-19-S), whereas normally distributed; while as median and 95% Confidence Interval (CI) when not normally distributed. After analyzing the continuous variables for skewness, kurtosis, normality distribution through the Shapiro-Wilk test, and the equality of variances by Levene test, parametric or non-parametric statistical tests were used when appropriate. Categorical variables (i.e., socio-demographic features, clinical and pregnancy-related variables) were presented in frequency (*n*) and percentage (%). Student's *t*-test for independent data and the non-parametric Mann-Whitney U-test for independent data were used, when appropriate, to compare the mean values of continuous variables among the two groups (pregnant vs. puerperal women) and between two groups (women with a significant EPDS score and women with a not significant EPDS score). The Chi-Square test was used to examine differences in the distribution of all categorical variables between two groups (pregnant vs. puerperal women) and between two groups (women with a significant EPDS score and women with a not significant EPDS score). One-way analysis of variance (ANOVA) or Kruskal-Wallis tests were used, where appropriate, to compare all continuous variables according to all socio-demographic and clinical categorical variables. Bivariate Pearson's correlations were used to investigate potential relationships between EPDS scores and other secondary continuous variables (CAS and FCV-19-S). A linear regression analysis was run to predict EPDS scores (dependent variable) from CAS (independent variable) and EPDS scores (dependent variable) from FCV-19-S (independent), after verifying all socio-demographic variables in both models as well. All the analyses were two-sided with a significance level settled at *p* < 0.05.

## Results

### Socio-demographic features of the sample

All socio-demographic characteristics of the included subjects are summarized in Table 1. A total of 184 women were consecutively assessed during the timeframe March 2020-March 2021. Among these, 85 patients gave written informed consent, agreed to provide their data for research purposes, and were recruited in the present study. After excluding those patients who subsequently decided to withdraw from the study (*N* = 6) and patients who did not fully fill out the questionnaires (*N* = 9), a final sample consisting of 70 subjects was finally included. The mean age was 34.8 years (SD = 5.8), without any significant differences between pregnant and postpartum women (*p* = 0.566). All women declared to be married or cohabiting with their partner, while 50% of the sample (*N* = 35) declared to be full-time employed and with an average middle-level of financial status declared (*N* = 61; 87.1%) (Table 1). Most women were assessed between January 2021 and March 2021 (*N* = 60; 85.7%), during the third trimester of their pregnancy (*N* = 36; 51.4%) and during the first postpartum trimester (*N* = 24; 34.3%). Most participants had a previous pregnancy (*N* = 44; 62.9%) and about 20% of participants (*N* = 14) declared to have experienced at least one miscarriage. Less than half of participants reported a current regular pregnancy course (*N* = 33; 47.1%) while most participants declared a desired pregnancy (*N* = 63; 90%) (Table 2).

**Table 1:** Socio-demographic characteristics of the sample.

	**Total Sample** **(*N =* 70)**	**Pregnant group** **(*N =* 41)**	**Postpartum group** **(*N =* 29)**	*p*-value^*^, ^**^
**Age** (years)	M *=* 34.8 (SD = 5.8)	M = 35.1 (SD = 6.0)	M = 34.3 (SD = 5.7)	^*^t(68) = 0.577 *p =* 0.566
**Nationality**				^**^
Italian From other European countries From non-European countries	60 (85.7%) 4 (5.7%) 6 (8.6%)	36 (87.8%) 2 (4.9%) 3 (7.3%)	24 (82.8%) 2 (6.9%) 3 (10.3%)	χ2(10) = 10.656 *p =* 0.385
**Marital status**	
Married/cohabiting	70 (100%)	41 (100%)	29 (100%)	n.d.
**Level of education**				^**^
Secondary School High school University degree Post-Degree	7 (10%) 26 (37.1%) 26 (37.1%) 11 (15.7%)	5 (12.2%) 14 (34.1%) 16 (39%) 6 (14.6%)	2 (6.9%) 12 (41.4%) 10 (34.5%) 5 (17.2%)	χ2(3) = 0.884 *p =* 0.829
**Employment status**				^**^
Student Housewife Employed Unemployed	3 (4.3%) 3 (4.3%) 52 (74.3%) 12 (17.1%)	2 (4.9%) 2 (4.9%) 29 (70.7%) 8 (19.5%)	1 (3.4%) 1 (3.4%) 23 (79.4%) 4 (13.8%)	χ2(4) = 1.444 *p =* 0.836
**Familiar nucleus**				^**^
Co-habitant partner/husband Co-habitant partner/husband and sons	35 (50%) 35 (50%)	21 (51.2%) 20 (48.8%)	14 (48.3%) 15 (51.7%)	χ2(1) = 0.059 *p =* 0.808
**Socio-economic status**				^**^
Low annual income Medium annual income High annual income	6 (8.6%) 61 (87.1%) 3 (4.3%)	5 (12.2%) 34 (82.9%) 2 (4.9%)	1 (3.4%) 27 (93.1%) 1 (3.4%)	χ2(2) = 1.799 *p =* 0.407

*M, mean; SD, standard deviation; n.d, not detected*.

* *Student's T-test;*

** *Pearson's χ2 test*.

**Table 2:** Obstetric-gynaecological characteristics of the sample.

	**Total sample (*N =* 70)**	**Pregnant group (*N =* 41)**	**Postpartum group (*N =* 29)**	*p*-value^*^
**Previous Pregnancy(ies)**				^*^
Current first pregnancy Current second pregnancy >2 previous pregnancies	26 (37.1%) 28 (40.0%) 16 (22.9%)	15 (36.6%) 15 (36.6%) 11 (26.8%)	11 (37.9%) 13 (44.8%) 5 (17.2%)	χ2(2) = 0.980 *p =* 0.613
**Previous miscarriage**	14 (20%)	8 (19.5%)	6 (20.7%)	^*^ χ2(1) = 0.015 *p =* 0.903
**Previous induced abortion**	3 (4.3%)	2 (4.9%)	1 (3.4%)	^**^ χ2(1) = 0.083 *p =* 0.629
Medical assisted procreation^**^	5 (7.1%)	4 (9.8%)	1 (3.4%)	^**^ χ2(1) = 1.004 *p =* 0.305
**Pregnancy course**				^*^
Regular without complications At-risk/with complications	33 (47.1%) 37 (52.9%)	14 (34.1%) 27 (65.9%)	19 (65.5%) 10 (34.5%)	χ(2) = 6.708 ***p** **=*** **0.015**
**LMD**	
2019 first semester 2019 second semester 2020 first semester 2020 second semester	2 (2.9%) 6 (8.6%) 45 (64.3%) 17 (24.3%)	1 (2.4%) 3 (7.3%) 23 (56.1%) 14 (34.1%)	1 (3.4%) 3 (10.3%) 22 (75.9%) 3 (10.3%)	n.v.
**EDD**	
2019 First semester 2019 Second semester 2020 First semester 2020 Second semester	4 (5.7%) 5 (7.1%) 59 (84.3%) 2 (2.9%)	1 (2.4%) 3 (7.3%) 25 (85.4%) 2 (4.9%)	3 (10.3%) 2 (6.9%) 24 (82.8%) 0 (0%)	n.v.
**Gestational and/or postpartum assessment period**	
1^st^ pregnancy trimester 2^nd^ pregnancy trimester 3^rd^ pregnancy trimester 1^st^ postpartum trimester 2^nd^ postpartum trimester 3^rd^/4^th^ postpartum trimester	2 (2.9%) 3 (4.3%) 36 (51.4%) 24 (34.3%) 1 (1.4%) 4 (5.7%)	2 (4.9%) 3 (7.3%) 35 (85.4%) 0 (0%) 0 (0%) 1 (2.4%)	0 (0%) 0 (0%) 1 (3.4%) 24 (82.8%) 1 (3.4%) 3 (10.3%)	n.v.

*EDD, estimated delivery date; LMD, last menstruation date; n.v., not valid. Significant p-values are in bold*.

* Pearson's χ2 test; ^**^ Fisher's exact test.

** *for the current pregnancy*.

### Clinical and psychopathological features of participants

Table 3 provides a summary of clinical and psychopathological data. Most of participants did not report any previous psychiatric history (*N* = 64; 91.4%), any previous psychiatric hospitalization (*N* = 69; 98.6%), any psychopharmacological therapy before pregnancy (*N* = 61; 87.1%) and/or during pregnancy (*N* = 61; 87.1%), either any current psychotherapy (*N* = 66; 94.3%) (Table 3).

**Table 3:** Clinical and psychopharmacological characteristics of the sample.

	**Total sample (*N =* 70)**	**Pregnant group** **(*N =* 41)**	**Postpartum group** **(*N =* 29)**	*p-*value^*^, ^**^
**Previous psychiatric history**	
Anxiety disorder Depressive disorder Bipolar disorder None	3 (4.3%) 2 (2.9%) 1 (1.4%) 64 (91.4%)	2 (4.9%) 2 (4.9%) 1 (2.4%) 36 (87.8%)	1 (3.4%) 0 (0%) 0 (0%) 28 (96.6%)	n.v.
**Previous psychiatric history**				^**^
None Yes	64 (91.4%) 6 (8.6%)	36 (87.8%) 5 (12.2%)	28(96.6%) 1 (3.4%)	χ2(1) = 1.635 *p =* 0.389
**Previous psychiatric hospitalization**				^**^
None Yes	69 (98.6%) 1 (1.4%)	40 (97.6%) 1 (2.4%)	29 (100%) 0 (0%)	χ2(1) = 0.707 *p =* 0.586
**Psychopharmacotherapy before pregnancy**	
None Antipsychotics Antidepressants Anxiolytics	61 (87.1%) 5 (7.1%) 2 (2.9%) 2 (2.9%)	34 (82.9%) 4 (9.8%) 2 (4.9%) 1 (2.4%)	27 (93.2%) 1 (3.4%) 0 (0%) 1 (3.4%)	n.v.
**Current psychopharmacotherapy**				^**^
None Yes	61 (87.1%) 9 (12.9%)	35 (85.4%) 7 (17.1%)	27 (93.1%) 2 (6.9%)	χ2(1) = 1.548 *p =* 0.289
**Current psychotherapy**				^**^
None Yes	66 (94.3%) 4 (5.7%)	38 (92.7%) 3 (7.3%)	28 (96.6%) 1 (3.4%)	χ2(1) = 0.465 *p =* 0.637
**EPDS**				^*^
<12 ≥ 12	38 (54.3%) 32 (45.7%)	20 (48.8%) 21 (51.2%)	18 (62.1%) 11 (37.9%)	χ2(1) = 1.209 *p =* 0.272
**EPDS, median**				^***^
**(95% CI)**	8.5 (7.7–10.2)	12.0 (7.8-11.1)	8.0 (6.3-10.2)	*U* = 509.5 *p =* 0.304
**FCV-19-S**				^*^
not clinically relevant clinically relevant	51 (72.9%) 19 (27.1%)	28 (68.3%) 13 (31.7%)	23 (79.3%) 6 (20.7%)	χ2(1) = 1.043 *p =* 0.307
**FCV-19-S, median**				^***^
**(95% CI)**	14.0 (13.5–16.4)	15.0 (13.8-18.2)	13.0 (11.8–15.1)	*U* = 482.0 *p =* 0.179
**CAS**				^**^
not clinically relevant clinically relevant	67 (95.7%) 3 (4.3%)	39 (95.1%) 2 (4.9%)	28 (96.6%) 1 (3.4%)	χ2(1) = 0.083 *p =* 0.629
**CAS, median**				^***^
**(95% CI)**	1.0 (1.1–2.4)	1.0 (0.8-2.8)	1.0 (0.7–2.6)	*U* = 622.0 *p =* 0.732

* *Pearson's χ2 test;*

** *Fisher's exact test;*

*** *U Mann-Whitney test*.

*M, mean; SD, standard deviation; n.v., not valid; EPDS, Edinburgh Postnatal Depression Scale; FCV-19-S, Fear of COVID-19; CAS, Coronavirus Anxiety Scale; CI, Confidence Interval*.

The mean total score at the EPDS was 9.0 (SD = 5.3), being experienced a clinically relevant perinatal depression (EPDS≥12) in 45.7% of the sample, without any significant differences between pregnant and puerperal women (*p* = 0.304) (Table 3).

**Figure 1:**
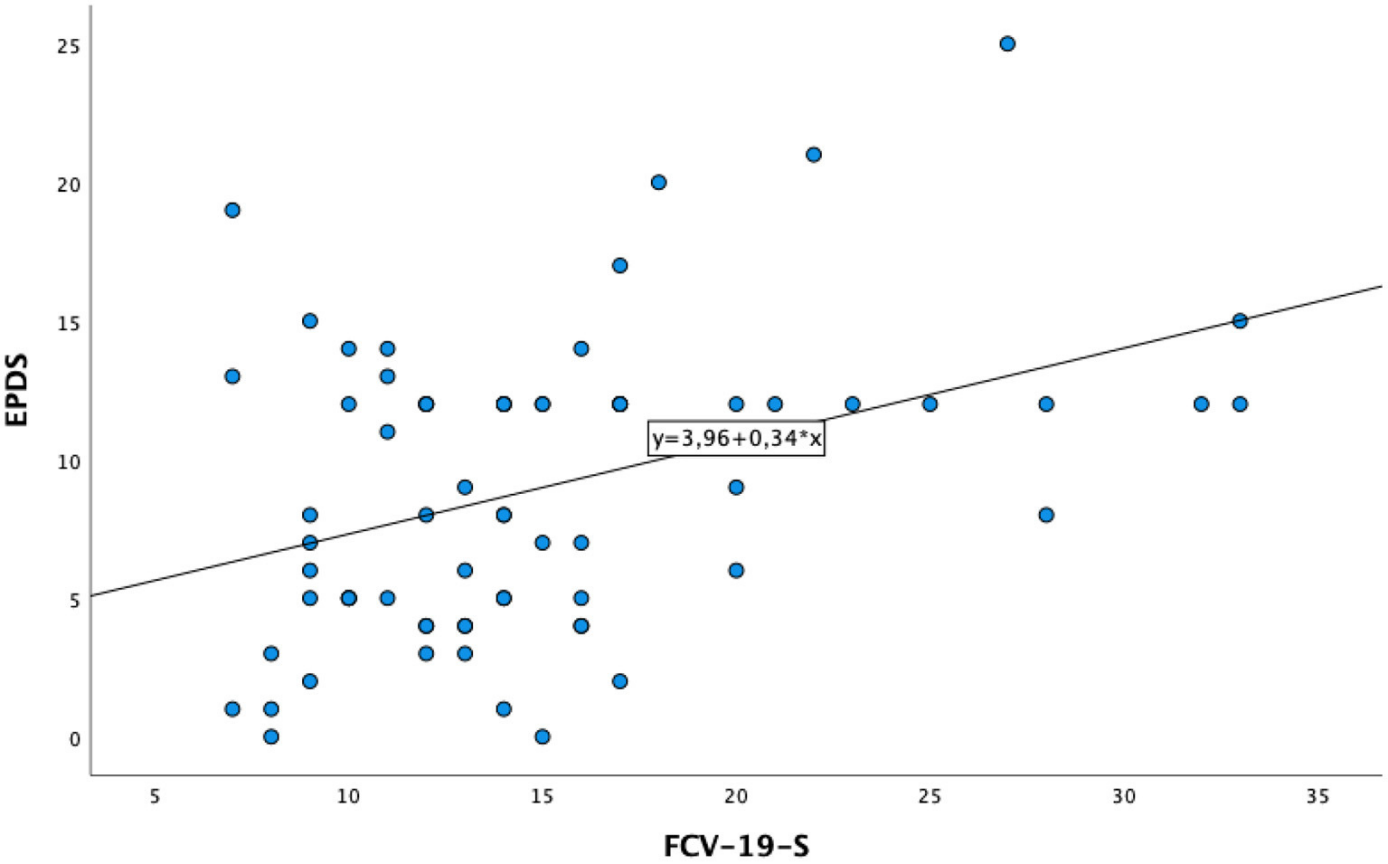
Linear regression model between FCV-19-S and EPDS.

The mean total score at FCV-19-S was 15.0 (SD = 6.2), with clinically relevant COVID-19-related fear (FCV-19-S≥16.86) experienced by 27.1% of participants, without any significant differences between pregnant and puerperal women (*p* = 0.179). Statistically significant higher FCV-19-S scores were found in women who had a previous psychiatric hospitalization (*p* = 0.029). Significant higher FCV-19-S scores were found in women with clinically relevant CAS total scores (*p* = 0.001) and clinically relevant EPDS total scores (*p* = 0.004) (Table 4). A positive correlation was found between FCV-19-S and EPDS (*r* = 0.390, *p* < 0.001) (Table 5). Linear regression analysis demonstrated that FCV-19-S scores statistically significantly predicted EPDS total scores [F(1,68) = 12.218, R^2^ = 0.152, *p* < 0.001] (Figure 1). No socio-demographic and/or clinical variables included in the regression model demonstrated to be predictive of EPDS scores.

**Table 4:** Psychopathological differences according to the EPDS screening.

	**EPDS– (*N =* 38)**	**EPDS+ (*N =* 32)**	*p*-value^*^
**FCV-19-S** **total score**, **median (95% CI)**	13.0 (11.6–14.2)	16.5 (14.8–20.0)	*U* = 849.0 ***p*** **=** **0.004**
**CAS** **total score**, **median (95% CI)**	1.0 (0.5–1.9)	2.0 (1.2–3.7)	*U* = 774.5 ***p** **=*** **0.040**

*EPDS+, with EPDS total score≥12; EPDS–, with EPDS total score <12; FCV+, with FCV-19-S≥16.86; FCV–, with FCV-19-S <16.86. Significant p-values are in bold*.

* *U Mann-Whitney test, two-tailed*.

*M, mean; SD, standard deviation; EPDS, Edinburgh Postnatal Depression Scale; FCV-19-S, Fear of COVID-19; CAS, Coronavirus Anxiety Scale; CI, Confidence Interval*.

**Table 5:** Linear regression models.

	**B**	**SE**	**Beta**	**t**	***p*-value**
**(constant)**	3.962	1.548		2.559	0.013
**FCV-19-S**	0.335	0.096	0.390	3.495	**<0.001**
	**B**	**SE**	**Beta**	**t**	***p*** **-value**
**(constant)**	7.798	0.697		11.185	<0.001
**CAS**	0.673	0.210	0.362	3.206	**0.002**

*EPDS, Dependent Variable. Significant p-values are in bold*.

^*^SE, standard error; CI, Confidence Interval; Significance at p <0.01 (two-tailored).

**Figure 2:**
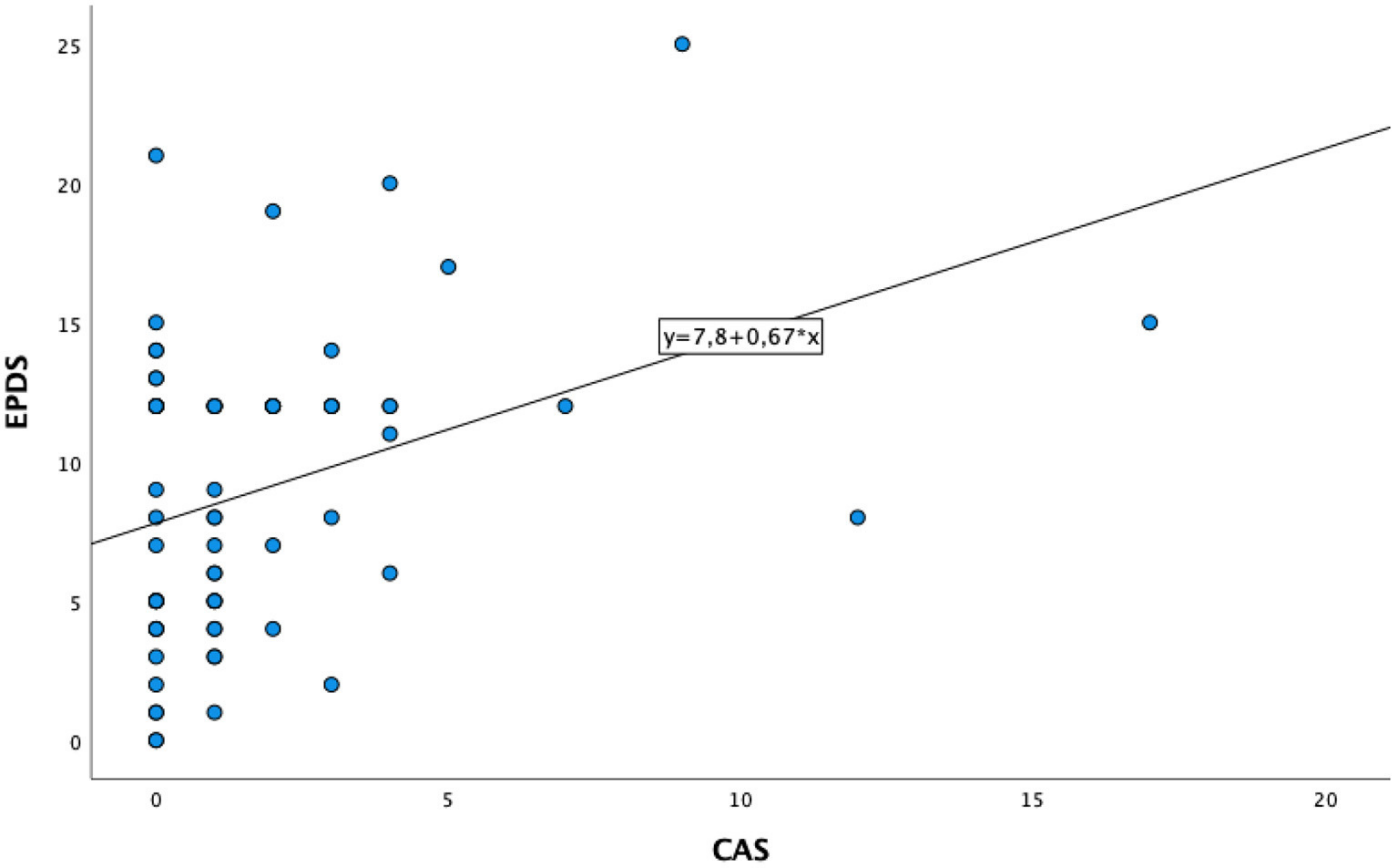
Linear regression model between CAS and EPDS.

The mean total score at CAS was 1.7 (SD = 2.8), with clinically relevant anxiety related to COVID-19 (CAS≥ 9) experienced by 4.3% of women, without any significant differences between pregnant and puerperal women (*p* = 0.732). Significant higher CAS scores were found in women who were positive for perinatal depression at EPDS (*p* = 0.040) (Table 4). A statistical trend with higher CAS scores was observed in those women with a previous psychiatric history of depressive episode(s) and/or major depressive disorder, compared to women without a previous psychiatric history (*p* = 0.054). A positive correlation was found between CAS and EPDS (*r* = 0.362, *p* < 0.001) and between CAS and FCV-19-S (*r* = 0.641, *p* < 0.001) (Table 5). Linear regression analysis demonstrated that CAS scores statistically significantly predicted EPDS total scores [F(1,68) = 10.278, R^2^ = 0.131, *p* = 0.002] (Figure 2). No socio-demographic and/or clinical variables included in the regression model demonstrated to be predictive of EPDS scores.

## Discussion

During the COVID-19 pandemic, pregnant and puerperal women worldwide reported increased levels of mental distress due to lack of access to healthcare, social isolation, sleep loss, feelings of fear and uncertainties (49–55). Overall, our sample reported a clinically relevant perinatal depression, as measured by EPDS, in 45.7% of the sample, with a higher rate, compared to previous international and national studies carried out before the COVID-19 pandemic (56–63). In fact, the prevalence of perinatal depression was estimated between 10–20% in non-Italian samples (59–62). While, in the few studies conducted to assess the Italian prevalence of perinatal depression, a highly variable prevalence was observed ranging from 1.6 to 26.6%, even though all of these studies were carried out before the COVID-19 pandemic indeed (42, 56–58, 64). Our findings are in line with previous published (both international and Italian) studies carried out during the COVID-19 pandemic which reported significantly higher depression rates in pregnant women than studies conducted before the pandemic, with a prevalence ranging from 30 to 43% (16, 18, 29, 31, 53, 65–71).

Although the effects of COVID-19 pandemic on perinatal mental health are still not fully investigated, pregnant and puerperal women represent indeed a particular vulnerable/at-risk population for developing mental health disorders, particularly during stressing situations, such as the current COVID-19 pandemic (72). Accordingly, our findings found that women, who have some mental distress related to the current COVID-19 outbreak, as measured by FCV-19-S and CAS scores, manifested clinically significant scores at EPDS. In particular, significant higher levels of COVID-19 fear were found in women who had a previous psychiatric hospitalization, by suggesting that women with a pre-existing psychiatric history may be more likely vulnerable to manifest fear of COVID-19 and, indirectly, manifest higher perinatal depressive levels compared to those without a previous psychiatric history. However, being our sample more represented by women without a psychiatric diagnosis, further larger studies specifically recruiting and comparing pregnant and postpartum women with and/or without a pre-existing psychiatric diagnosis should be carried out to better investigate this hypothesis. Moreover, our findings reported a significant positive correlation between fear of COVID-19 and COVID-19-related anxiety levels, as well as between fear of COVID-19 and perinatal depression levels, as already documented in previous studies (73–75). Furthermore, our findings documented a significant positive correlation between COVID-19-related anxiety and perinatal depression levels, as already demonstrated in previous studies (76, 77). In fact, the fear of contagion and for the health of the child, the difficulty in promptly accessing to health care system due to the COVID-19 restrictive measures, as well as the poor availability in being supported by own partner and/or family members during the hospitalization for the delivery may represent all factors which may have determined increased depressive and anxious symptoms in women during the peripartum period (67, 70, 72, 78). The increased levels of COVID-19 anxiety seems to be related to specific concerns about the impact of the COVID-19 on maternal health, fetal/neonatal health, vertical transmission of COVID-19 infection from mother to fetus and worries regarding the potential separation and social distancing from family and social relationships during the perinatal period due to quarantine measures (67, 79, 80). In fact, the most critical fears and worries experienced by pregnant and postpartum women regard the possibility of family members to be not present during the perinatal period, during the hospitalization, labor and childbirth while restriction policies in hospital settings are in place (31, 81).

Moreover, most participants of our study did not report any previous psychiatric history and/or psychiatric hospitalization and/or any psychopharmacological treatment and/or psychological support before pregnancy. Therefore, our findings suggest that increased levels of perinatal depression may be experienced during the COVID-19 pandemic, more likely due to isolation and quarantine experience, also by pregnant and puerperal women, independently by pre-existing psychiatric disorders. Moreover, our sample is more representative of perinatal period comprising the third trimester of pregnancy and the first postpartum trimester, hence, one could argue that our findings might potentially reflect the effect of the COVID-19 pandemic during this period and that higher levels of perinatal depression observed in our sample might be due an effect dependent on the perinatal stage, as already documented in previous studies (31, 82). In fact, according to these studies, the risk of negative psychological consequences during the COVID-19 pandemic may be increased especially in pregnant women in their third trimester who foresee delivery during the pandemic, as they may experience elevated stress and anxiety due to the potential adverse outcomes on the fetus and the infant (31, 79, 82). Despite a larger longitudinal study by Mei et al. (30) found that the gestational trimester had no correlation with depression, anxiety and stress rates. Therefore, further studies should assess and investigate the perinatal stage variable on perinatal depression, anxiety and stress.

Despite the abovementioned promising findings, the present study has several limitations. Firstly, the cross-sectional study design and the small sample size may limit the generalizability of the findings and may not be fully representative of the full peripartum period, being mainly recruited women at their third trimester of pregnancy and during their first postpartum trimester. The attrition rate between women assessed and women included was indeed mainly due to expressed worry by pregnant and puerperal women recruited during the COVID-19 pandemic to find some relevant COVID-19-related psychopathology and the lack of time to fill out all questionnaires administered (particularly among puerperal women). The lack of a control group constituted by not-pregnant women, coming from both a clinical and not-clinical sample, may not allow the comparability of the findings and may not adequately evaluate the gender-effect on the development of higher depressive scores, independently by the pregnancy and/or postpartum period during the COVID-19 pandemic. Moreover, another issue is the lack of a control group constituted by males, for instance including the partners of recruited women and/o coming from the general population may not allow to discriminate whether the observed effect of COVID-19-related anxiety and fear may really impacting on the perinatal depression due to the gender effect or rather the COVID-19-related psychopathological burden in the vulnerable population of pregnant and/or puerperal women. Secondly, our sample is constituted mainly by women without a previous psychiatric history which may not allow us to completely evaluate the differential impact of the COVID-19 pandemic on pregnant and/or postpartum women with a previous psychiatric disease and compare them with those with a negative psychiatric history. Thirdly, although we collected several socio-demographic and clinical variables in our sample, we did not find that none of these socio-demographic and/or clinical variables demonstrated to be significant predictors of EPDS scores. However, these findings could be mainly due to the small sample size here recruited. Therefore, a larger study recruiting also women with more heterogeneous socio-demographic features could allow researchers to better understand whether a specific socio-demographic and/or clinical profile could represent a predictor of EPDS scores during the COVID-19 pandemic. Moreover, even though the administered assessment tools here chosen, demonstrated to be valid and highly reliable measures of COVID-19-related fear and anxiety symptomatology, some limitations of these self-report questionnaires should be carefully considered and discussed when we interpret our findings. For instance, while some studies reported no gender differences on the FCV-19S (45), other studies reported higher FCV-19S scores in females compared to males (4, 48). Similarly, CAS scores were found to be higher in females compared to males in the development and psychometric study of the tool (48, 83). Finally, our sample is represented by women without a previous and/or a current COVID-19 infection, hence, our findings may not completely evaluate whether the pregnant women with COVID-19 infection may be more or less likely to develop a perinatal depression compared with pregnant women without COVID-19 infection and/or not pregnant women with COVID-19 infection.

Therefore, further research directions performing longitudinal and case-control studies with larger sample sizes, including as potential variables the concomitant COVID-19 infection during pregnancy and/or postpartum period, should be conducted to better evaluate whether the gender-effect might explain the increased levels of depression in pregnant and/or postpartum women during the COVID-19 pandemic, as already reported in previous Italian studies which observed more severe psychological symptoms during the COVID-19 pandemic reported by females compared to males in Italian population (84–86). In fact, “caution is needed when reporting opinions or data coming from cross-sectional studies, especially in the absence of proper controls for lockdown” (87). Moreover, further studies should investigate how experiencing feelings of fear and anxiety related to the COVID-19 might determine increased levels of depression, independently by the pregnancy and/or postpartum period in women compared to men. Moreover, one should better investigate whether women with a previous psychiatry history may be more or less likely to develop increased levels of perinatal depression compared to women without a previous psychiatry history during the COVID-19 pandemic, independently by the variable to be infected with COVID-19 or not. Overall, our findings may indeed address clinicians to better evaluate and early identify those women at high-risk to develop perinatal depression during the COVID-19 pandemic, by investigating their levels of fear and perceived anxiety/distress due to the COVID-19 situation for preventive, screening and monitoring strategies. Finally, one could argue that a possible strategy which may help to improve screening activities could be implementing a smartphone-based screening tool consisting of CAS and FCV-19-S questionnaires which could be periodically and virtually administered to those pregnant and puerperal women to indirectly identify those at-risk to develop a perinatal depression in order to propose a psychological and/or psychiatric support (whereas necessary).

## Conclusion

The COVID-19 pandemic and subsequent isolation, quarantine and lockdown represent a risk factor for pregnant and postpartum women who may experience a deprivation of their normal sources of family and social support and, hence, experience increased psychological distress. Our findings might address clinicians and politicians towards tailored clinical and policy implications to be implemented in the perinatal women, such as providing dedicated spaces and/or support figures, trained specifically on perinatal mental health consequences due to the COVID-19 pandemic and related restrictions, if possible. Trained mental health professionals can help women feel less isolated while facing the labor and postpartum period, within hospitals, during the COVID-19 pandemic, by offering psychoeducational interventions on perinatal mental health as well as COVID-19 and perinatal mental health issues. Moreover, implementing public mental health policies to allow a direct and indirect screening for perinatal depression during the COVID-19 pandemic.

## Data availability statement

The raw data supporting the conclusions of this article will be made available by the authors, without undue reservation.

## Ethics statement

Ethical review and approval was not required for the study on human participants in accordance with the local legislation and institutional requirements. The patients/participants provided their written informed consent to participate in this study.

## Author contributions

LO and UV: conceptualization. LO: methodology, formal analysis, and writing—review and editing. LO, AM, and SP: data curation and collection. SP and LO: writing—original draft preparation. LO, VS, and SP: investigation. VS: visualization. UV: supervision. All authors contributed to the article and approved the submitted version.

## Conflict of interest

The authors declare that the research was conducted in the absence of any commercial or financial relationships that could be construed as a potential conflict of interest. The handling editor MS declared a past co-authorship/collaboration with one of the authors LO.

## Publisher's note

All claims expressed in this article are solely those of the authors and do not necessarily represent those of their affiliated organizations, or those of the publisher, the editors and the reviewers. Any product that may be evaluated in this article, or claim that may be made by its manufacturer, is not guaranteed or endorsed by the publisher.
